# Comparative efficacy and safety of COVID-19 vaccines in phase III trials: a network meta-analysis

**DOI:** 10.1186/s12879-023-08754-3

**Published:** 2024-02-21

**Authors:** Xiaodi Wu, Ke Xu, Ping Zhan, Hongbing Liu, Fang Zhang, Yong Song, Tangfeng Lv

**Affiliations:** 1 Medical School of Nanjing University, Nanjing, 210000 China; 2 Department of Respiratory and Critical Care Medicine, Jinling Hospital, Affiliated Hospital of Medical School, Nanjing University, Nanjing, 210000 China

**Keywords:** SARS-CoV-2, COVID-19, Vaccine, Efficacy, Network Meta-Analysis

## Abstract

**Background:**

Over a dozen vaccines are in or have completed phase III trials at an unprecedented speed since the World Health Organization (WHO) declared COVID-19 a pandemic. In this review, we aimed to compare and rank these vaccines indirectly in terms of efficacy and safety using a network meta-analysis.

**Methods:**

We searched Embase, MEDLINE, and the Cochrane Library for phase III randomized controlled trials (RCTs) from their inception to September 30, 2023. Two investigators independently selected articles, extracted data, and assessed the risk of bias. Outcomes included efficacy in preventing symptomatic severe acute respiratory syndrome coronavirus 2 (SARS-CoV-2) infection and the incidence of serious adverse events (SAEs) according to vaccine type and individual vaccines in adults and elderly individuals. The risk ratio and mean differences were calculated with 95% confidence intervals using a Bayesian network meta-analysis.

**Results:**

A total of 25 RCTs involving 22 vaccines were included in the study. None of vaccines had a higher incidence of SAEs than the placebo. Inactivated virus vaccines might be the safest, with a surface under the cumulative ranking curve (SUCRA) value of 0.16. BIV1-CovIran showed the highest safety index (SUCRA value: 0.13), followed by BBV152, Soberana, Gam-COVID-Vac, and ZF2001. There were no significant differences among the various types of vaccines regarding the efficacy in preventing symptomatic SARS-CoV-2 infection, although there was a trend toward higher efficacy of the mRNA vaccines (SUCRA value: 0.09). BNT162b2 showed the highest efficacy (SUCRA value: 0.02) among the individual vaccines, followed by mRNA-1273, Abdala, Gam-COVID-Vac, and NVX-CoV2373. BNT162b2 had the highest efficacy (SUCRA value: 0.08) in the elderly population, whereas CVnCoV, CoVLP + AS03, and CoronaVac were not significantly different from the placebo.

**Conclusions:**

None of the different types of vaccines were significantly superior in terms of efficacy, while mRNA vaccines were significantly inferior in safety to other types. BNT162b2 had the highest efficacy in preventing symptomatic SARS-CoV-2 infection in adults and the elderly, whereas BIV1-CovIran had the lowest incidence of SAEs in adults.

**Supplementary Information:**

The online version contains supplementary material available at 10.1186/s12879-023-08754-3.

## Introduction

There have been over 600 million confirmed cases of coronavirus disease (COVID-19) and over 6 million worldwide deaths by the end of 2022 since the onset of the COVID-19 pandemic [1]. The pandemic has significantly impacted healthcare and socio-economic development worldwide. The most prevalent clinical features of COVID-19 include fever, cough, and dyspnea [2]. While most cases are mild, the elderly and those with underlying diseases are at high risk of severe COVID-19. Moreover, some people also experience long-term effects after recovery. Novel oral antivirals such as molnupiravir, fluvoxamine, and paxlovid [3] are still under development, and heteropathy is believed to be the main clinical treatment. Therefore, vaccination is the first and most important step in stopping the spread of COVID-19 and reducing the social burden.

Vaccines can be divided into five categories according to their principles of antigen generation and production processes: inactivated virus vaccines, mRNA vaccines, DNA vaccines, viral vector vaccines, and protein subunit vaccines. Each type has certain advantages. Inactivated viral vaccines containing intact spike proteins and other proteins protect against viral variants by inducing a broader immune response [4]. mRNA and DNA vaccines are rapid and cost-effective platforms that can simulate natural infections by synthesizing endogenous proteins to induce a strong immune response [5]. Viral vector vaccines are characterized by robust immunogenicity, the absence of adjuvants, and long-term storage without freezing [6]. Protein subunits vaccines can produce robust and durable antibody responses and are expected to be safer because they do not utilize genetic materials [7].

Vaccine efficacy (VE) data are primarily obtained from phase III randomized controlled trials (RCTs). Previous studies have compared the efficacy and safety of vaccines using multiple post-hoc pairwise comparisons in meta-analyses [8–10]. In June 2021, a meta-analysis was conducted for eight Phase III RCTs encompassing four vaccine types [8]. The study indicated that all vaccine types exhibited good preventive effects against COVID-19, accompanied by an elevated risk of overall adverse events in the vaccinated groups. However, these studies did not compare multiple vaccines administered under identical conditions [8]. A network meta-analysis (NMA) provides a methodological approach to simultaneously compare vaccines through a common comparator (placebo) since there are no head-to-head clinical studies directly comparing the relative efficacy and safety of COVID-19 vaccines. In April 2021, the first published NMA of four Phase III RCTs showed that the vaccine exhibited different efficacies to prevent COVID-19: BNT162b2 ≥ mRNA-1273 > Gam-COVID-Vac > AZD1222 [11]. Subsequently, Rotshild et al. reported no statistical differences among vaccines in the preventive effect against severe COVID-19 of the elderly [12]. The latest NMA evaluation of the efficacy of 16 vaccines (October 2022) revealed that BNT126b2 conferred the highest protection against symptomatic severe acute respiratory syndrome coronavirus 2 (SARS-CoV-2) infection [13].

This study aimed to integrate the latest published data from Phase III RCTs to compare the efficacy and safety of COVID-19 vaccines in adult populations. The efficacy of COVID‑19 vaccines was also conducted to prevent symptomatic disease among the elderly. This manuscript was written following the PRISMA-NMA checklist [14].

## Methods

### Search strategy and selection criteria

A systematic search was performed in PubMed, EMBASE, the Cochrane Library, medRxiv, and SSRN from their inception to Sep 30, 2023 for COVID-19 vaccine studies. The search included the following keywords and subject terms: “COVID-19,” “SARS-CoV-2,” “vaccines,” “efficacy,” “safety” and “clinical trial”. Details regarding the search strings for the different databases are provided in Table S1.

The PICOS design approach was used to formulate the study eligibility criteria:

#### Population

Subjects who participated in clinical trials related to COVID-19 vaccines, aged > 18 years, and without a prior history of SARS-CoV-2 infection or COVID-19 vaccination.

#### Intervention

The intervention was to complete the COVID-19 vaccination according to the design plan. We selected the optimal administration regimen approved by the relevant agencies as the only intervention when a vaccine contained multiple regimens.

#### Comparison

Placebo or COVID-19 vaccines.

#### Outcome

The efficacy outcomes included the incidence of laboratory-confirmed (RT-PCR-positive) symptomatic SARS-CoV-2 infection. Safety outcomes included serious adverse events (SAEs).

#### Study design

Phase III RCTs with full-text publications were included.

### Data extraction and quality assessment

Two investigators (XDW and KX) independently selected the articles and extracted data according to the title, abstract, full reports, and supplementary materials. All discrepancies were resolved by consensus between two other authors of the study (HBL and PZ). Data were extracted in three parts: study characteristics (date of publication, author, phase, sample size, trial country, and study design), baseline demographic characteristics (sex ratio and age range), vaccine characteristics (vaccine type, company, adjuvant, injection interval, and concentration), and outcomes (definition, and follow-up time). The quality of individual studies was evaluated using RoB2 (version 2 of the Cochrane tool for assessing the risk of bias in randomized trials) [15]. The five assessed sources of risk of bias were randomization process, deviations from intended intervention, missing outcome data, measurement of the outcome, and selection of the reported result.

### Outcomes

The primary outcomes included type-specific efficacy and safety of COVID-19 vaccines in adults. Vaccines were divided into five categories: inactivated viral vaccines, mRNA vaccines, DNA vaccines, viral vector vaccines, and protein subunit vaccines. The secondary outcomes included the efficacy and safety of individual vaccines in adults, type-specific efficacy of COVID-19 vaccines in the elderly, and the efficacy of individual vaccines in the elderly.

VE was evaluated by comparing the difference in the number of laboratory-confirmed (RT-PCR-positive) symptomatic SARS-CoV-2 infection cases commencing 7–28 days after the last dose of the investigational product between the experimental and control groups.

Safety outcomes were evaluated as the number of participants that reported SAEs throughout the study period. Analysis of SAEs included all participants who received at least one dose. SAEs were defined in accordance with the ICH-GCP as any untoward medical contingency that resulted in death, was life-threatening, requiring hospitalization, or resulted in persistent or significant disability or incapacity at any dose, regardless of whether they were considered as associated with vaccination [16]. Safety analysis of the vaccines was limited to adults only, as no clinical research provided SAE data for the elderly.

### Data synthesis and statistical analysis

An NMA only including indirect comparisons was conducted to compare and rank the COVID-19 vaccines in terms of efficacy and safety in the absence of trials directly comparing the two COVID-19 vaccines. Heterogeneity was initially assessed using the Cochrane Q test and I² statistics were calculated. A random-effects model was used when I² was greater than 50% and a fixed-effects model was used when I² was below 50%. Possible causes of heterogeneity were explored through sensitivity analysis. The transitivity underlying NMA was subjectively evaluated by comparing key clinical features. Inconsistency was not evaluated since no study directly compared the two vaccines. The risk ratio (RR) was chosen for the outcomes with a corresponding 95% confidence interval (95% CI) to determine the effect size. The model was run based on simulations of 20,000 iterations in the framework of the Bayesian theory with each of the four chains after a burn-in of 5,000 using Markov chain Monte Carlo (MCMC) techniques with Gibbs sampling. Model fit was ensured using trace plots, density plots with bandwidth, and Brooks-Gelman-Rubin diagnostic plots. Network diagrams were used to present the networks for the models, and the outcomes of pairwise comparisons were presented in the corresponding tables. The surface under the cumulative ranking curve (SUCRA) was calculated to summarize probability values and rank the interventions measured on a scale of 0 (best) to 1 (worst) [17]. Potential publication bias of the included studies was evaluated using a funnel plot and Egger’s test. All analyses were conducted using the “gemtc” package and “rjags” package that interfaces with JAGS 4.3.0 in R x64 4.0.3 (R Foundation for Statistical Computing, Vienna, Austria) [18–20].

## Results

**Fig. 1:**
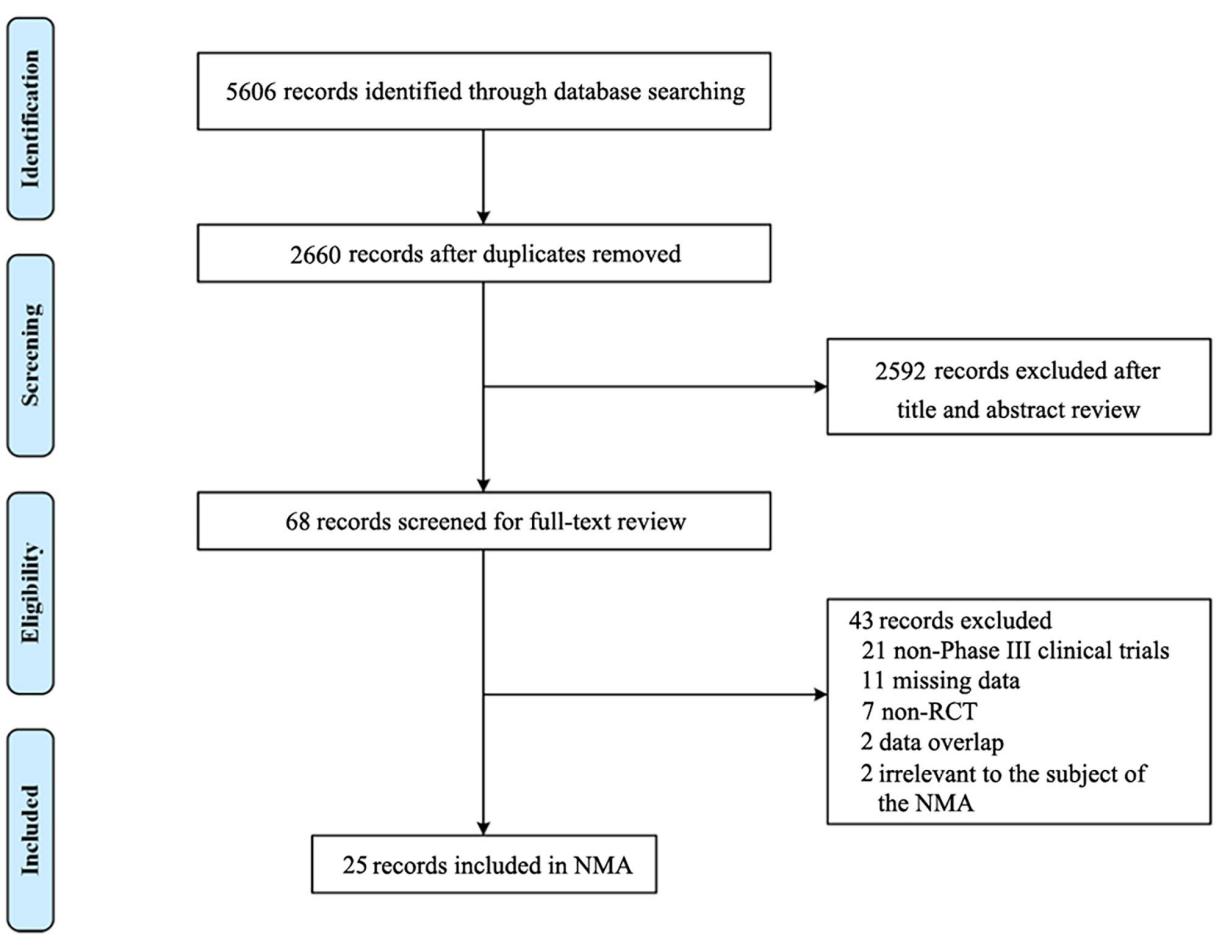
Flowchart of study selection

A total of 5606 records were identified by the search, with 24 published and one unpublished Phase III RCT [21–45] involving 22 vaccines eventually included in the NMA (Fig. 1). Two of the search results included a small number of individuals under the age of 18 years [29, 31], and another study included Phase I/II/III RCTs of AZD1222 vaccines [34]. These three studies were included in the NMA to ensure a sufficient number of samples. None of the included studies directly compared two different vaccines. In total, 915,370 participants were included, and more than 50% were randomly assigned for vaccination. Study characteristics and raw data are summarized in Table 1 and S2. A comparison of basic features, including outcome definition and participant characteristics (age, sex, and race) is presented in Table S3 and Figure S1. There was no evidence of violation of the transitivity assumption. Among these articles, studies with some concerns accounted for 36%, but there were no serious risks of bias according to the RoB2 (Figure S2).

**Table 1:** Characteristics of the clinical studies included in the network meta-analysis

Vaccine Type	Study ID	Vaccine Name	Phase	Study designs	Trial Country	Study Period	Dose	Injection Interval	Concentration	Participants		
										Total(T/C)	Mean Age (years)	Male (%)
Inactivated vaccine	Ella, 2021 NCT04641481	BBV152	III	Double-blind RCT	India	Nov 16, 2020-Jan 7, 2021	2	28	6 µg	8471/8502	40.1	67.1
	Kaabi, 2021 NCT04510207	WIBP-CorV (WIV04)	III	Double-blind RCT	The United Arab Emirates, Bahrain	Jul 16, 2020-Dec 31, 2020	2	21	5 µg	12,743/12,737	36.1	84.4
	Kaabi, 2021 NCT04510207	BBIBP-CorV (HB02)	III	Double-blind RCT	The United Arab Emirates, Bahrain	Jul 16, 2020-Dec 31, 2020	2	21	4 µg	12,726/12,737	-	84.4
	Tanriover, 2021 NCT04582344	CoronaVac	III	Double-blind RCT	Turkey	Sept 14, 2020-Mar 16, 2021	2	14	3 µg	6559/3470	-	57.8
	Palacios, 2021 NCT04456595	CoronaVac	III	Double-blind RCT	Brazil	Jul 21, 2020-Dec 16, 2020	2	14	3 µg	4953/4870	39.5	35.8
	Fadlyana,2021 NCT04508075	CoronaVac	III	Observer-blinded RCT	Indonesia	Aug 11, 2020-Oct 21, 2020	2	14	3 µg	798/804	35.5	64.6
	Khairullin, 2022 NCT04691908	QazCovid-in	III	Single-blind RCT	Kazakhstan	Dec 25, 2020- Jul 11, 2021	2	21	5 mg	2400/600	-	51
	Mohraz, 2023 IRCT20201202049567N3	BIV1-CovIran	III	Double-blind RCT	Iran	May 16, 2021-Jul 15, 2021	2	28	5 µg	13,335/6665	38.3	65.4
mRNA vaccine	Sahly, 2021 NCT04470427	mRNA-1273	III	Observer-blinded RCT	US	Jul 27, 2020-Oct 23, 2020	2	28	100 µg	14,287/14,164	51.4	52.6
	Polack, 2020 NCT04368728	BNT162b2	III	Observer-blinded RCT	US, Argentina, Brazil	Jul 27, 2020-Nov 14, 2020	2	21	30 µg	17,411/17,511	-	50.6
	Kremsner, 2022 NCT04652102	CVnCoV	III	Observer-blinded RCT	Europe, Latin America	Dec 11, 2020-Apr 12, 2021	2	28	12 µg	12,851/12,211	43	54.9
DNA vaccine	Khobragade, 2022 CTRI/2021/01/030416	ZyCoV-D	III	Double-blind RCT	India	Jan 16, 2021-Jun 23, 2021	3	28	2 mg	12,350/12,320	36.5	67.11
Viral vector vaccine	Logunov, 2021 NCT04530396	Gam-COVID-Vac (Sputnik V)	III	Double-blind RCT	Russia	Sept 7, 2020-Nov 24, 2020	2	21	10¹¹	14,964/4902	45.3	55.1
	Sadoff, 2022 NCT04505722	Ad26.COV2.S	III	Double-blind RCT	Argentina, Brazil, Chile, Colombia, Mexico, Peru, South Africa, US	Sept 21, 2020-Jul 9, 2021	1	N/A	5 × 10¹^10^	19,514/19,544	-	54.9
	Voysey, 2021 NCT04324606 NCT04400838 NCT04536051 NCT04444674	AZD1222 (ChAdOx1 nCoV-19)	I/II	Single-blind RCT	UK, cov001	Apr 23, 2020-Nov 6, 2020	2	28–84	3·5–6·5 × 10¹^10^	7201/7179	-	39.5
			II/III		UK, cov002							
			III		Brazil, cov003							
			I/II	Double-blind RCT	South Africa, cov005							
	Falsey, 2021 NCT04516746	AZD1222 (ChAdOx1 nCoV-19)	III	Double-blind RCT	US, Chile, Peru	Aug 28, 2020-Jan 15, 2021	2	14	5 × 10¹^10^	17,662/8550	50.2	55.6
	Halperin, 2022 NCT04526990	Ad5-nCoV	III	Double-blind RCT	Argentina, Chile, Mexico, Pakistan, Russia	Sept 22, 2020-Jan 15, 2021	1	N/A	5 × 10¹^10^	14,591/14,586	37.8	70.8
Protein subunit vaccine	Heath, 2021 2020-004123-16	NVX-CoV2373	III	Observer-blinded RCT	UK	Sept 28, 2020- Nov 28, 2020	2	21	5 µg	7020/7019	-	51.6
	Dunkle, 2022 NCT04611802	NVX-CoV2373	III	Observer-blinded RCT	US, Mexico	Dec 27, 2020- Feb 18, 2021	2	21	5 µg	17,312/8140	-	51.8
	Bravo, 2022 NCT04672395	SCB-2019	II/III	Double-blind RCT	Belgium, Brazil, Colombia, Philippines, South Africa	Mar 24, 2021-Aug 10, 2021	2	21	30 µg	6251/6104	31.1	55
	Dai, 2022 NCT04646590	ZF2001	III	Double-blind RCT	Uzbekistan, Indonesia, Pakistan, Ecuador, China	Dec 12, 2020-Dec 15, 2021	3	30	25 µg	12,625/12,568	36.8	67.5
	Tabarsi, 2022 NCT05005559	SpikoGen	III	Double-blind RCT	Iran	Aug 7, 2021-Nov 2021	2	21	25 µg	12,657/4219	33.1	56.6
	Bernal, 2023 RPCEC00000359	Abdala	III	Double-blind RCT	Cuba	Mar 22, 2021-Jun 2021	3	14	50 µg	24,146/24,144	48.9	47.6
	Ryzhikov, 2023 NCT04780035	EpiVacCorona	III	Double-blind RCT	Russia	Nov 27, 2020-Aug 31, 2021	2	21	225 ± 45 µg	2253/746	48.4	51.9
	Mostafavi, 2023 IFV/COR/09	Soberana	III	Double-blind RCT	Iran	Apr 26, 2021-Sep 25, 2021	3	28	25–50µg	4340/1081	39.7	59.8
	Hager, 2022 NCT04636697	CoVLP + AS03	III	Double-blind RCT	Argentina, Brazil, Canada, Mexico, UK, US	Mar 15, 2021-Sept 2, 2021	2	21	3.75 µg	12,074/12,067	32.8	50.9

RCT, random clinical trial; N/A, not applicable; -, missing data

### Comparative efficacy and safety of different types of vaccines in adults

**Fig. 2:**
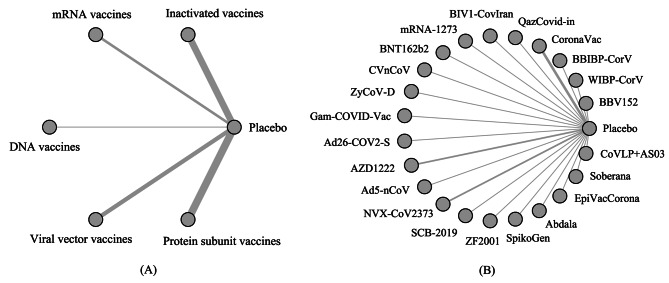
Network diagram (**A**) Network diagram of type-specific efficacy for adults. (**B**) Network diagram of individual vaccine efficacy for adults. The thickness of the lines is proportional to the number of trials comparing every pair of treatments

We explored the differences in efficacy and safety between different types of vaccines using NMA. Vaccines were divided into five categories: inactivated viral vaccines, mRNA vaccines, DNA vaccines, viral vector vaccines, and protein subunit vaccines. Star-shaped network diagrams of the primary outcomes are shown in Fig. 2(A) and S3.

**Fig. 3:**
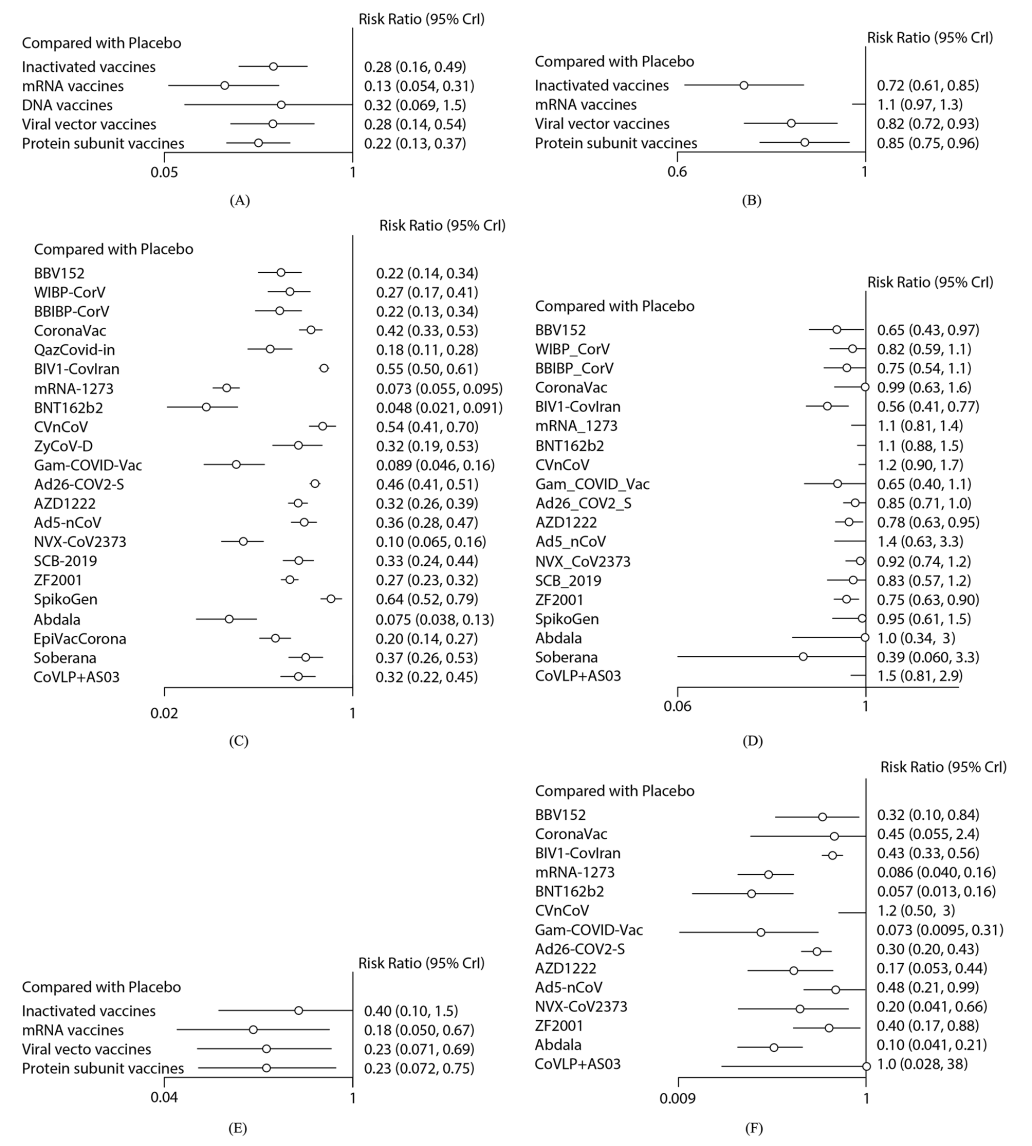
Forest plot of intervention compared to the placebo in the network meta-analysis. RR, risk ratio; CI, confidence interval. (**A**) Forest plot of the efficacy of different vaccine types in adults. (**B**) Forest plot of the safety of different vaccine types in adults. (**C**) Forest plot of individual vaccine efficacy for adults. (**D**) Forest plot of individual vaccine safety for adults. (**E**) Forest plot of efficacy of different vaccine types in the elderly. (**F**) Forest plot of individual vaccine efficacy for the elderly

The inactivated viral, mRNA, viral vector, and protein subunit vaccines were predictably more effective than the placebo in terms of efficacy (25 RCTs involving 22 vaccines), with RRs ranging between 0.13 (95% CI [0.05, 0.31]) for mRNA vaccines and 0.28 [0.16, 0.49] for inactivated viral vaccines (Fig. 3(A)). The DNA vaccines (0.32 [0.07, 1.5]) were not statistically significant compared with the placebo. There were no significant differences between the various types of vaccines in the indirect pairwise comparisons (Table S4), although there was a trend in the mRNA vaccines for the lowest risk of symptomatic disease, with the lowest SUCRA value of 0.09 (Table S5).

In terms of safety (21 RCTs involving 19 vaccines), none of vaccines had a higher incidence of SAEs than the placebo (Fig. 3(B)). The inactivated virus vaccine ranked first, with a SUCRA value of 0.04, whereas the mRNA vaccine ranked last, with a SUCRA value of 0.98 (Table S6). There was a significant difference in the side effect rates between mRNA vaccines and other vaccine types in the indirect pairwise comparisons (Table S7). Funnel plots and Egger’s tests revealed asymmetry in VE and no asymmetry in vaccine safety (Figure S4).

### Comparative efficacy and safety of individual vaccines in adults

Network diagrams are shown in Fig. 2(B) and S5. In terms of efficacy (25 RCTs involving 22 vaccines), all 22 vaccines were more effective than the placebo, with RRs ranging between 0.05 [0.02, 0.09] for BNT162b2 and 0.64 [0.52, 0.79] for SpikoGen (Fig. 3(C)). According to the outcome of pairwise comparisons (Table S8) and SUCRA value (Table S9), BNT162b2 had the highest efficacy (SUCRA value: 0.02), followed by mRNA-1273, Abdala, Gam-COVID-Vac, and NVX-CoV2373. The efficacy of SpikoGen was the lowest, with a SUCRA of 0.94.

In terms of safety (21 RCTs involving 19 vaccines), none of the vaccines had a higher incidence of SAEs than the placebo (Fig. 3(D)). BIV1-CovIran had the highest probability of being the vaccine with the lowest incidence of SAEs (SUCRA value: 0.1), followed by BBV152, Soberana, Gam-COVID-Vac, and ZF2001. In contrast, the safety of CoVLP + AS03 was the lowest, with a SUCRA value of 0.89. There were no statistically significant differences between most of the vaccines. Details of the pairwise comparisons and SUCRA values are shown in Tables S10 and S11.

### Comparative efficacy of different types of vaccines in the elderly population

Data on efficacy in the elderly population were retrieved from 15 RCTs involving 14 vaccines. Vaccines are divided into four categories: inactivated virus vaccines, mRNA vaccines, viral vector vaccines, and protein subunit vaccines. The definition of the elderly population slightly differed across the included studies, ranging from 50 to 65 years. Star-shaped network diagram is shown in Figures S6.

The mRNA, viral vector, and protein subunit vaccines were predictably more effective than the placebo, with RRs ranging from 0.18 [0.05, 0.67] for mRNA vaccines and 0.23 [0.07, 0.75] for protein subunit vaccines (Fig. 3(E)). The inactivated virus vaccine (0.4 [0.1, 1.5]) was not statistically significant compared to the placebo. There were no significant differences between the various types of vaccines in the indirect pairwise comparisons (Table S12), although there was a trend in the mRNA vaccine for the lowest risk of symptomatic disease, with the lowest SUCRA value of 0.24 (Table S13). Funnel plots and Egger’s tests indicated no publication bias (Figure S7).

### Comparative efficacy of individual vaccines in the elderly population

Star-shaped network diagram is shown in Figures S8. 11 of the 14 vaccines had good preventive effects against COVID-19 compared with the placebo, with RRs ranging between 0.06 [0.01, 0.16] for BNT162b2 and 0.48 [0.21, 0.99] for Ad5-nCoV (Fig. 3(F)). CVnCoV, CoVLP + AS03, and CoronaVac were interpreted as having no differences from the placebo. BNT162b2 had the lowest SUCRA value of 0.08, with the highest probability of being the most effective vaccine for the elderly, followed by Gam-COVID-Vac and mRNA-1273, whereas CVnCoV had the lowest probability, with the highest SUCRA value of 0.92. Details of the SUCRA values and pairwise comparisons are shown in Tables S14 and S15.

### Additional analyses

Sensitive analyses were performed after excluded trials with a follow-up time of less than 2 months. 18 RCTs were included in analyses. The results were stable and were similar to the main analysis after excluding 7 trials (Table S16). In addition, sensitivity analyses were performed after excluded the unpublished study, and the results are robust.

## Discussion

This study was based on 25 RCTs that included 915,370 patients randomly assigned to receive 22 vaccines or a placebo. This project updates and extends previous research and is the most comprehensive NMA to compare the efficacy of COVID-19 vaccines in preventing symptomatic disease and the incidence of SAEs in adults and the elderly.

In terms of safety, mRNA vaccines may increase SAEs versus the placebo, although this result was not statistically significant. Similar trends were described in an earlier meta-analysis of 11 trials [46]. Our results provided the following rankings according to RR in the indirect comparison: inactivated vaccines ≥ viral vector vaccines ≥ protein subunit vaccines > mRNA vaccines. This is unsurprising given the high safety of inactivated vaccines since no viral genetic material is involved. In addition to SAEs, inactivated vaccines have the lowest risk of local or systemic adverse events following immunization [47]. The ranking of individual vaccines was generally consistent with the vaccine type. BIV1-CovIran, an inactivated vaccine, had the lowest incidence of SAEs. Notably, most included studies did not specifically exclude patients with symptomatic COVID-19 from SAE, which may have affected the accuracy of the above ranking.

In terms of efficacy, all vaccine types versus placebo significantly prevented symptomatic SARS-CoV-2 infection, but the 95% CI for DNA vaccines indicated no effect. In the indirect comparison, our results provided the following ranking according to the RR: mRNA vaccines ≥ protein subunit vaccines ≥ viral vector vaccines ≥ inactivated vaccines ≥ DNA vaccines. The 95% CI for all vaccine types was compatible with no effect, although the RR values were significant. One possible explanation for the excellent efficacy of mRNA vaccines is the production of a fully functional protein through cellular translational machinery, which induces powerful and durable immunity against the coronavirus [48]. An earlier NMA compared nine vaccines to prevent symptomatic SARS-CoV-2 infection, based on the results of Phase III RCTs up to August 1, 2021 [12]. BNT162b2 had the highest efficacy, followed by mRNA‑1273, Gam‑COVID‑Vac, NVX‑CoV2373, CoronaVac, BBIBP-CorV, WIBP-CorV, and Ad26.COV2.S [12]. Similarly, one recent NMA reported that BNT126b2 conferred the highest protection, followed by mRNA-1273, Gam‑COVID‑Vac and NVX-CoV2373 [13]. In line with previous evidence, we ranked BNT162b2 with the highest efficacy, followed by mRNA-1273, Abdala, Gam-COVID-Vac, and NVX-CoV2373. We also found that BNT162b2 and mRNA-1273 mRNA vaccines performed best in preventing symptomatic COVID-19, while CVnCoV ranked lower. A possible explanation is that approximately 85% of COVID-19 cases in the CVnCoV trial were caused by variants that might alter VE owing to the increased transmissibility and evasion of neutralizing humoral immunity [49]. In addition, 12 µg mRNA contained in CVnCoV may be insufficient to elicit a protective immune response compared to 30 µg in BNT162b2 and 100 µg in mRNA-1273.

We found that BNT162b2 had the highest efficacy in terms of the efficacy in preventing symptomatic SARS-CoV-2 infection in the elderly population. This was consistent with the conclusion of an earlier study [47]. CVnCoV, CoVLP + AS03, and CoronaVac were interpreted as having no difference from the placebo, possibly owing to an insufficient absolute number of events in the short follow-up duration. In fact, the VE of CoronaVac in the real world has reached 66.6% in individuals aged > 60 [50]. In addition to the elderly, the impact of vaccines on children is gradually emphasized. Recently published Phase III clinical trials show that mRNA-1273 [51–53], BNT162b2 [54], and BBIBP-CorV [55] are safe in populations younger than 18 years and trigger an immune response no less than that in young people. There is a lack of large-scale clinical trials to support the active use of COVID-19 vaccines for other populations, such as pregnant women, immunodeficient patients, and people that were previously exposed to SARS-CoV-2.

Our review has some limitations; the above results should be cautiously interpreted since inconsistencies were not assessed in the absence of trials that directly compared the two COVID-19 vaccines. The transitivity assumption underlying the NMA was evaluated by comparing key clinical features, including participant characteristics (age, sex, and race), and outcome assessment (definition and measurement). However, there are some differences in the research background and protocols, such as vaccine dose and different SARS-CoV-2 variants, which might lead to deviations in analytical results. Furthermore, vaccines face great challenges in terms of increasing the diversity of variants, and the ranking of VE can change. Booster vaccines are necessary to prevent SARS-CoV-2 variant infections and provide durable immunity. These data suggest that homologous and heterologous booster vaccines have an acceptable safety profile and heterologous boosting may be more immunogenic than homologous boosting [56]. Our conclusion aims to provide a primary reference for vaccine selection. However, other important factors such as the prevention of severe COVID-19, long-term side effects, and economic considerations should also be considered practical scenarios.

## Conclusions

Our study is the most comprehensive NMA exploring the efficacy and safety of type-specific and individual COVID-19 vaccines based on the latest data. Our analysis showed that BIV1-CovIran inactivated vaccine had the lowest incidence of SAEs in adults, and BNT162b2 mRNA vaccine had the highest efficacy in preventing symptomatic SARS-CoV-2 infections in adults and the elderly population.

### Electronic supplementary material

Below is the link to the electronic supplementary material.

Supplementary Material 1

Supplementary Material 2

## Data Availability

All data generated or analyzed during this study are included in this published article and its additional information files.

## Abbreviations

CI: Confidence interval
MCMC: Markov chain Monte Carlo
NMA: Network meta-analysis
RCTs: Randomized controlled trials
RR: Risk ratio
SAEs: Serious adverse events
SARS-CoV-2: Severe acute respiratory syndrome coronavirus 2
SUCRA: Surface under the cumulative ranking curve
VE: Vaccine efficacy
