# Supplementary material for: Comparative efficacy and safety of COVID-19 vaccines in phase III trials: a network meta-analysis
Source: BMC Infect Dis. 2024 Feb 21;24:234. doi: 10.1186/s12879-023-08754-3 (PMC10880292; doi:10.1186/s12879-023-08754-3)
Supplement: Supplementary file 1 — Supplementary Material 1 [file 12879_2023_8754_MOESM1_ESM.pdf]

Checklist of Items to Include When Reporting a Systematic Review Involving a Network Meta-analysis

Section/Topic	Item No*	Checklist item†	Reported on Page Number/Line Number	Reported on Section/Paragraph
**TITLE**				
Title	1	Identify the report as a systematic review *incorporating a network meta-analysis (or related form of meta-analysis)*.	Page 1/Line 2	Title page/Title
**ABSTRACT**				
Structured summary	2	Provide a structured summary including, as applicable:<br>Background: main objectives<br>Methods: data sources; study eligibility criteria, participants, and interventions; study appraisal; and *synthesis methods, such as network meta-analysis*.<br>Results: number of studies and participants identified; summary estimates with corresponding confidence/credible intervals; *treatment rankings may also be discussed. Authors may choose to summarize pairwise comparisons against a chosen treatment included in their analyses for brevity*.<br>Discussion/Conclusions: limitations; conclusions and implications of findings.<br>Other: primary source of funding; systematic review registration number with registry name.	Page 2-3/Line 23-46	Abstract
**INTRODUCTION**				
Rationale	3	Describe the rationale for the review in the context of what is already known, *including mention of why a network meta-analysis has been conducted*.	Page 3-4/Line 49-79	Introduction
Objectives	4	Provide an explicit statement of questions being addressed, with reference to participants, interventions, comparisons, outcomes, and study design (PICOS).	Page 4/Line 80-82	Introduction
**METHODS**				
Protocol and registration	5	Indicate whether a review protocol exists and if and where it can be accessed (e.g., Web address); and, if available, provide registration information, including registration number.	N/A<br>No review protocol exists	N/A
Eligibility criteria	6	Specify study characteristics (e.g., PICOS, length of follow-up) and report characteristics (e.g., years considered, language, publication status) used as criteria for eligibility, giving rationale.<br>*Clearly describe eligible treatments included in the treatment network, and note whether any have been clustered or merged into the same node (with justification).*	Page 5/Line 89-98	Methods/Search strategy and selection criteria
Information sources	7	Describe all information sources (e.g., databases with dates of coverage, contact with study authors to identify additional studies) in the search and date last searched.	Page 4/Line 85-86	Methods/Search strategy and selection criteria

Search	8	Present full electronic search strategy for at least one database, including any limits used, such that it could be repeated.	Page 4/Line 86-88	Methods/Search strategy and selection criteria; Supplementary
Study selection	9	State the process for selecting studies (i.e., screening, eligibility, included in systematic review, and, if applicable, included in the meta-analysis).	Page 5/Line 100-101	Methods/Data extraction and quality assessment
Data collection process	10	Describe method of data extraction from reports (e.g., piloted forms, independently, in duplicate) and any processes for obtaining and confirming data from investigators.	Page 5/Line 100-102	Methods/Data extraction and quality assessment
Data items	11	List and define all variables for which data were sought (e.g., PICOS, funding sources) and any assumptions and simplifications made.	Page 5/Line 102-105; Page 6/Line 110-122	Methods/Data extraction and quality assessment; Methods/Outcomes
Geometry of the network	**S1**	Describe methods used to explore the geometry of the treatment network under study and potential biases related to it. This should include how the evidence base has been graphically summarized for presentation, and what characteristics were compiled and used to describe the evidence base to readers.	Page 7/Line 134-135	Methods/Data synthesis and statistical analysis
Risk of bias within individual studies	12	Describe methods used for assessing risk of bias of individual studies (including specification of whether this was done at the study or outcome level), and how this information is to be used in any data synthesis.	Page 5/Line 105-108	Methods/Data extraction and quality assessment
Summary measures	13	State the principal summary measures (e.g., risk ratio, difference in means). *Also describe the use of additional summary measures assessed, such as treatment rankings and surface under the cumulative ranking curve (SUCRA) values, as well as modified approaches used to present summary findings from meta-analyses*.	Page 6/Line 130-131; Page 7/Line 135-137	Methods/Data synthesis and statistical analysis
Planned methods of analysis	14	Describe the methods of handling data and combining results of studies for each network meta-analysis. This should include, but not be limited to:<br>*Handling of multigroup trials;*<br>*Selection of variance structure;*<br>*Selection of prior distributions in Bayesian analyses; and Assessment of model fit.*	Page 6-7/Line 131-134	Methods/Data synthesis and statistical analysis
Assessment of inconsistency	**S2**	Describe the statistical methods used to evaluate the agreement of direct and indirect evidence in the treatment network(s) studied. Describe efforts taken to address its presence when found.	Page 6/Line 129-130	Methods/Data synthesis and statistical analysis
Risk of bias across studies	15	Specify any assessment of risk of bias that may affect the cumulative evidence (e.g., publication bias, selective reporting within studies).	Page 7/Line 137-138	Methods/Data extraction and quality assessment
Additional analyses	16	Describe methods of additional analyses if done, indicating which were prespecified. This may include, but not be limited to, the following:<br>Sensitivity or subgroup analyses;<br>Meta-regression analyses;<br>*Alternative formulations of the treatment network; and*<br>*Use of alternative prior distributions for Bayesian analyses (if applicable)*.	Page 6/Line 127-128	Methods/Data synthesis and statistical analysis

**RESULTS‡**				
Study selection	17	Give numbers of studies screened, assessed for eligibility, and included in the review, with reasons for exclusions at each stage, ideally with a flow diagram.	Page 7/Line 142-143	Results/Figure 1
Presentation of network structure	**S3**	Provide a network graph of the included studies to enable visualization of the geometry of the treatment network.	Page 8/Line 155 and 170; Page 9/Line 186 and 194	Results/Figure 2; Supplementary Appendix/Figures S3,5,6,8
Summary of network geometry	**S4**	Provide a brief overview of characteristics of the treatment network. This may include commentary on the abundance of trials and randomized patients for the different interventions and pairwise comparisons in the network, gaps of evidence in the treatment network, and potential biases reflected by the network structure.	Page 7/Line 143-151	Results
Study characteristics	18	For each study, present characteristics for which data were extracted (e.g., study size, PICOS, follow-up period) and provide the citations.	Page 7/Line 147-148	Results/Table 1
Risk of bias within studies	19	Present data on risk of bias of each study and, if available, any outcome level assessment.	Page 7-8/Line 149-151	Results/Figure S2
Results of individual studies	20	For all outcomes considered (benefits or harms), present, for each study: 1) simple summary data for each intervention group, and 2) effect estimates and confidence intervals. *Modified approaches may be needed to deal with information from larger networks*.	Page 7/Line 147-148	Results/Table S2
Synthesis of results	21	Present results of each meta-analysis done, including confidence/credible intervals. *In larger networks, authors may focus on comparisons versus a particular comparator (e.g., placebo or standard care), with full findings presented in an appendix. League tables and forest plots may be considered to summarize pairwise comparisons*. If additional summary measures were explored (such as treatment rankings), these should also be presented.	Page 7-10/Line 152-200	Results
Exploration for inconsistency	**S5**	Describe results from investigations of inconsistency. This may include such information as measures of model fit to compare consistency and inconsistency models, P values from statistical tests, or summary of inconsistency estimates from different parts of the treatment network.	N/A<br>Inconsistency was not evaluated as no study directly compared two	N/A
Risk of bias across studies	22	Present results of any assessment of risk of bias across studies for the evidence base being studied.	Page 8/Line 167-168; Page 9/Line 192	Results/Figures S4 and S7
Results of additional analyses	23	Give results of additional analyses, if done (e.g., sensitivity or subgroup analyses, meta-regression analyses, *alternative network geometries studied, alternative choice of prior distributions for Bayesian analyses*, and so forth).	Page 10/Line 201-205	Results/Additional analyses

**DISCUSSION**				
Summary of evidence	24	Summarize the main findings, including the strength of evidence for each main outcome; consider their relevance to key groups (e.g., health care providers, researchers, and policymakers).	Page 10-12/Line 207-247	Discussion
Limitations	25	Discuss limitations at study and outcome level (e.g., risk of bias), and at review level (e.g., incomplete retrieval of identified research, reporting bias). *Comment on the validity of the assumptions, such as transitivity and consistency. Comment on any concerns regarding network geometry (e.g., avoidance of certain comparisons).*	Page 12/Line 248-259	Discussion
Conclusions	26	Provide a general interpretation of the results in the context of other evidence, and implications for future research.	Page 12-13/Line 261-264	Conclusions
**FUNDING**				
Funding	27	Describe sources of funding for the systematic review and other support (e.g., supply of data); role of funders for the systematic review. This should also include information regarding whether funding has been received from manufacturers of treatments in the network and/or whether some of the authors are content experts with professional conflicts of interest that could affect use of treatments in the network.	Page 13/Line 285-286	Declarations/Funding

* Boldface indicates new items to this checklist.

† Text in italics indicates wording specific to reporting of network meta-analyses that has been added to guidance from the PRISMA statement.

‡ Authors may wish to plan for use of appendices to present all relevant information in full detail for items in this section.

Please leave this space alone as it will be supplemented by the editorial office when needed.
