# Supplementary material for: Comparative efficacy and safety of COVID-19 vaccines in phase III trials: a network meta-analysis
Source: BMC Infect Dis. 2024 Feb 21;24:234. doi: 10.1186/s12879-023-08754-3 (PMC10880292; doi:10.1186/s12879-023-08754-3)
Supplement: Supplementary file 2 — Supplementary Material 2 [file 12879_2023_8754_MOESM2_ESM.pdf]

Supplementary Appendix

Table S1. Search strategy……………………………………………………………………………...…..3

Table S2. Raw data of included studies……………………………………………………………..……….4

Table S3. Definition, time horizon, and follow-up time of efficacy outcome………..…………….….……….6

Figure S1. Comparison of demographic information of included studies……………………………..............8

Figure S2. Overall risk of bias assessment using Version 2 of the Cochrane tool for assessing risk of bias in randomized trials…………………………………………………………………………………..…………9

Table S4. Comprehensive comparisons for type-specific efficacy……………………………………....……10

Table S5. SUCRA value of various types of vaccines in the network meta-analysis of type-specific efficacy…....10

Figure S3. Network diagram of type-specific safety……………………………………………...…………10

Table S6. SUCRA value of various types of vaccines in the network meta-analysis of type-specific safety……...11

Table S7. Comprehensive comparisons for type-specific safety……………………………..………….……11

Figure S4. Funnel plot and Egger's test of vaccine efficacy and safety………………………………………....11

Table S8. Comprehensive comparisons for efficacy of individual vaccines in adults……………………………12

Table S9. SUCRA value of various vaccines in the network meta-analysis of vaccine efficacy in adults……….13

Figure S5. Network diagram of individual vaccine safety in adults……………………………………………..13

Table S10. Comprehensive comparisons for safety of individual vaccines in adults……………………………..14

Table S11. SUCRA value of various vaccines in the network meta-analysis of vaccine safety in adults…………15

Figure S6. Network diagram of type-specific efficacy in the elderly……………………….…………………..…15

Table S12. Comprehensive comparisons for type-specific efficacy in the elderly……………………………...…16

Table S13. SUCRA value of various types of vaccines in the network meta-analysis of type-specific efficacy in the elderly……………………………………………………………………………………………...…16

Figure S7. Funnel plot and Egger's test of vaccine efficacy in the elderly……………………..………………….…16

Figure S8. Network diagram of individual vaccine efficacy in the elderly…………………………………….…17

Table S14. SUCRA value of various vaccines in the network meta-analysis of vaccine efficacy in the elderly…………………………………………………………………………...…………………..…17

Table S15. Comprehensive comparisons for efficacy of individual vaccines in the elderly………………………18

Table S16. Results of network meta-analysis after excluding trials with a follow-up time of less than 2 months…19

Table S1. Search strategy

**PubMed**	
#1	(COVID-19[MeSH Terms]) OR (2019 novel coronavirus disease[Title/Abstract]) OR (2019-nCoV[Title/Abstract]) OR (SARS-CoV-2[Title/Abstract])
#2	(vaccine[MeSH Terms])
#3	(efficacy[Title/Abstract]) OR (safety[Title/Abstract]) OR (side effects[Title/Abstract])
#4	(randomized controlled trial[MeSH Terms]) OR (controlled clinical trial[Title/Abstract]) OR (randomized[Title/Abstract]) OR (placebo[Title/Abstract]) OR (randomly[Title/Abstract]) OR (trial[Title/Abstract]) OR (groups[Title/Abstract])
#5	#1 AND #2 AND #3 AND #4

Table S2. Raw data of included studies

Study ID	Vaccine Name	Symptomatic COVID-19 (adults)		Serious Adverse Events (adults)		Symptomatic COVID-19 (elderly)	
		**Intervention** (cases/total)	**Control** (cases/total)	**Intervention** (cases/total)	**Control** (cases/total)	**Intervention** (cases/total)	**Control** (cases/total)
Ella,2021	BBV152	24/8471	106/8502	39/12879	60/12874	5/893	16/965
Kaabi,2021	WIBP-CorV	26/12743	95/12737	64/13464	78/13453	-	-
Kaabi,2021	BBIBP-CorV	21/12726	95/12737	59/13471	78/13453	-	-
Tanriover,2021	CoronaVac	9/6559	32/3470	6/6646	5/3568	-	-
Palacios,2021	CoronaVac	85/4953	168/4870	33/6202	31/6194	2/212	4/207
Fadlyana,2021	CoronaVac	7/798	18/804	-	-	-	-
Khairullin,2022	QazCovid-in	31/2400	43/600	-	-	-	-
Mohraz,2023	BIV1-CovIran	758/12945	688/6456	83/13335	74/6665	105/2234	116/1064
Sahly,2021	mRNA-1273	55/14287	744/14164	104/15162	98/15184	9/14287	100/14164
Polack,2020	BNT162b2	8/17411	162/17511	126/21621	111/21631	3/7500	48/7543
Kremsner,2022	CVnCoV	82/12851	145/12211	82/19783	66/19746	12/1319	9/1180
Khobragade, 2022	ZyCoV-D	20/12350	61/12320	-	-	-	-
Logunov,2021	Gam-COVID-Vac	13/14094	47/4601	45/16427	23/5435	2/1611	8/533
Sadoff,2022	Ad26.COV2.S	495/19400	1082/19398	223/3356	265/3380	36/6764	120/6762

Voysey,2021	AZD1222	74/7201	197/7179	108/12282	127/11962	-	-
Falsey,2021	AZD1222	73/17662	130/8550	101/8771	53/3201	5/3696	14/1812
Halperin,2022	Ad5-nCoV	77/14591	211/14586	14/18363	10/18354	10/1323	21/1347
Heath,2021	NVX-CoV2373	10/7020	96/7019	41/7569	41/7570	1/1953	9/1957
Dunkle,2022	NVX-CoV2373	14/17312	63/8140	169/19729	94/9853	2/2048	2/946
Bravo,2022	SCB-2019	52/6251	155/6104	49/15064	59/15064	-	-
Dai,2022	ZF2001	158/12625	580/12568	199/14448	264/14425	8/704	20/722
Tabarsi,2022	SpikoGen	247/9998	119/3069	71/12657	25/4219	-	-
Bernal,2023	Abdala	11/19689	142/19674	7/24146	7/24144	7/9927	66/9874
Ryzhikov,2023	EpiVacCorona	58/2148	89/649	-	-	-	-
Mostafavi,2023	Soberana	75/4800	51/1200	3/4790	2/1197	-	-
Hager,2022	CoVLP+AS03	40/12074	125/12067	24/12036	16/12040	1/62	1/65

-, missing data

Table S3. Definition, time horizon, and follow-up time of efficacy outcome

Study ID	Vaccine Name	Definition of symptomatic COVID-19	Time horizon of efficacy assessment	Follow-up time*
Ella, 2021	BBV152	At least one symptoms condition for 1-2 days or more: (1) at least two consecutive days with one or more specific symptoms (cough, newly developed taste or smell disorders, shortness of breath or dyspnea); (2) with two or more non-specific symptoms (fever, chills, sore throat, fatigue, nasal congestion or runny nose, body pain, muscle pain, headache, nausea or vomiting, diarrhoea.	At least 14 days after the last dose	99 days
Kaabi, 2021	WIBP-CorV		At least 14 days after the last dose	77 days
Kaabi, 2021	BBIBP-CorV		At least 14 days after the last dose	77 days
Palacios, 2021	CoronaVac		At least 14 days after the last dose	About 76 days
Fadlyana, 2021	CoronaVac		At least 14 days after the last dose	75 days
Mohraz, 2023	BIV1-CovIran		At least 14 days after the last dose	83 days
Sahly, 2021	mRNA-1273		At least 14 days after the last dose	169 days
Bravo, 2022	SCB-2019		At least 14 days after the last dose	68 days
Tabarsi, 2022	SpikoGen		At least 14 days after the last dose	55 days
Bernal, 2023	Abdala		At least 14 days after the last dose	33 days
Mostafavi,2023	Soberana		At least 14 days after the last dose	142 days
Khairullin, 2022	QazCovid-in	At least one of the following symptoms for 1-2 days or more: fever or chills; cough; dyspnoea; fatigue; muscle or body pain; headache; new loss of sense of smell or change in taste; sore throat; nasal congestion or rhinorrhoea; nausea or vomiting; and diarrhoea.	At least 14 days after the last dose	About 159 days
Tanriover, 2021	CoronaVac		At least 14 days after the last dose	62 days
Polack, 2020	BNT162b2		At least 7 days after the last dose	About 91 days
Kremsner, 2022	CVnCoV		At least 15 days after the last dose	48.2 days

Sadoff, 2022	Ad26.COV2.S		At least 14 days after the last dose	120 days
Voysey, 2021	AZD1222		At least 14 days after the last dose	About 90 days
Falsey, 2021	AZD1222		At least 15 days after the last dose	46 days
Halperin, 2022	Ad5-nCoV		At least 14 days after the last dose	38 days
Heath, 2021	NVX-CoV2373		At least 28 days after the last dose	62 days
Dunkle, 2022	NVX-CoV2373		At least 7 days after the last dose	About 60 days
Dai, 2022	ZF2001		At least 7 days after the last dose	About 180 days
Hager, 2022	CoVLP+AS03		At least 7 days after the last dose	45 days
Ryzhikov, 2023	EpiVacCorona	-	At least 21 days after the last dose	About 138 days
Khobragade, 2022	ZyCoV-D	-	At least 28 days after the last dose	About 62 days
Logunov, 2021	Gam-COVID-Vac	-	At least 7 days after the last dose	20 days

* The follow-up time is calculated from 7 to 28 days after the last dose.

-, missing data.

Figure S1. Comparison of demographic information of included studies. (A) Sex (B) Race. Data missing: Ella, 2021; Kaabi, 2021; Tanriover, 2021; Fadlyana, 2021; Khobragade,2022; Kremsner, 2022; Khairullin, 2022; Mohraz, 2023; Ryzhikov, 2023; Mostafavi, 2023; Tabarsi, 2022 (C) Mean age. Data missing: Heath, 2021; Sadoff, 2022; Polack, 2020; Tanriover, 2021; Voysey, 2021; Dunkle, 2021; Khairullin, 2022

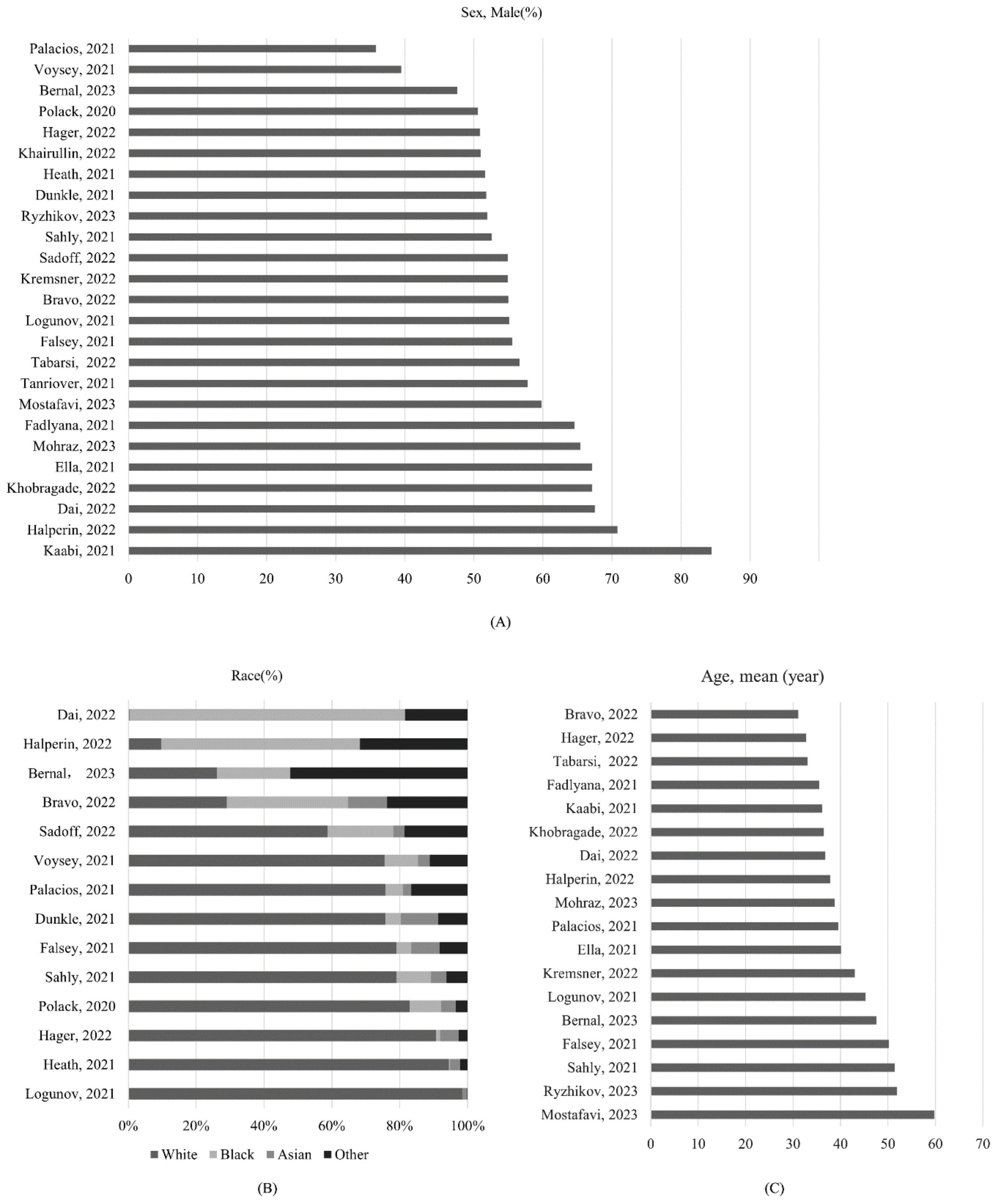

Figure S2. Overall risk of bias assessment using Version 2 of the Cochrane tool for assessing risk of bias in randomized trials. (A) Overall risk of bias assessment using the Cochrane tool. (B) Risk of bias assessment by individual trials

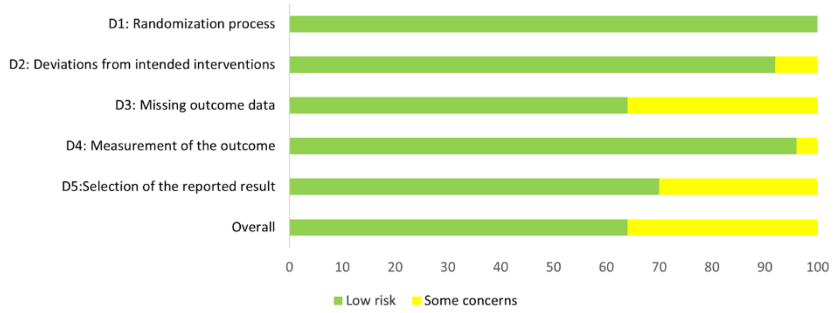

(A)

Study ID	D1	D2	D3	D4	D5	Overall
Ella,2021	+	+	+	+	+	+
Kaabi,2021	+	+	?	+	?	?
Tanriover,2021	+	+	?	+	?	?
Palacios,2021	+	+	+	+	+	+
Sahly,2021	+	+	+	+	+	+
Polack,2020	+	+	+	+	+	+
Logunov,2021	+	+	?	+	?	?
Sadoff,2022	+	+	+	+	+	+
Voysey,2021	+	?	?	+	?	?
Heath,2021	+	+	?	+	+	?
Dunkel,2021	+	+	?	+	+	?
Dai,2022	+	+	+	+	+	+
Fadlyana,2021	+	+	?	+	?	?
Falsey,2021	+	+	+	+	+	+
Hager,2022	+	+	+	+	+	+
Halperin,2022	+	+	+	+	+	+
Khobragade,2022	+	+	?	+	?	?
Kremsner,2022	+	+	+	+	+	+
Bravo,2022	+	+	+	+	+	+
Khairullin,2022	+	?	?	+	+	?
Mohraz, 2023	+	+	+	+	+	+
Tabarsi, 2022	+	+	+	+	+	+
Bernal, 2023	+	+	+	+	+	+
Ryzhikov, 2023	+	+	?	+	+	+
Mostafavi, 2023	+	+	+	+	+	+

+ Low risk

? Some concerns

(B)

Table S4. Comprehensive comparisons for type-specific efficacy.

**Placebo**					
	**DNA vaccine**				
3.53 (2.05, 6.05)		**Inactivated vaccine**			
3.53 (1.84, 6.89)			**Viral vector vaccine**		
4.44 (2.69, 7.39)				**Protein subunit vaccine**	
7.61 (3.22, 18.54)					**mRNA vaccine**

Data are RRs (95% CI) in the column-defining intervention compared with the row-defining intervention. RR higher than 1 favors the row-defining intervention. To obtain RRs for comparisons in the opposite direction, reciprocals should be taken. Results without statistical significance were deleted from the table.

Table S5. SUCRA value of various types of vaccines in the network meta-analysis of type-specific efficacy.

Vaccine Type	SUCRA value
Placebo	0.99
DNA vaccine	0.55
Inactivated vaccine	0.52
Viral vector vaccine	0.51
Protein subunit vaccine	0.35
mRNA vaccine	0.09

Figure S3. Network diagram of type-specific safety. The thickness of the lines is proportional to the number of trials comparing every pair of treatments.

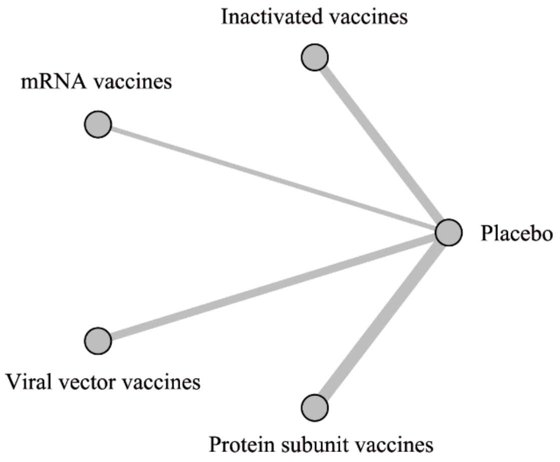

Table S6. SUCRA value of various types of vaccines in the network meta-analysis of type-specific safety.

Vaccine Type	SUCRA value
mRNA vaccine	0.98
Placebo	0.76
Protein subunit vaccine	0.4
Viral vector vaccine	0.31
Inactivated vaccine	0.04

Table S7. Comprehensive comparisons for type-specific safety.

**mRNA vaccine**				
	**Placebo**			
1.34 (1.08, 1.63)	1.17 (1.04, 1.32)	**Protein subunit vaccine**		
1.38 (1.13, 1.70)	1.22 (1.07, 1.38)		**Viral vector vaccine**	
1.57 (1.25, 1.97)	1.38 (1.17, 1.63)			**Inactivated vaccine**

Data are RRs (95% CI) in the column-defining intervention compared with the row-defining intervention. RR higher than 1 favors the row-defining intervention. To obtain RRs for comparisons in the opposite direction, reciprocals should be taken. Results without statistical significance were deleted from the table.

Figure S4. Funnel plot and Egger's test of vaccine efficacy and safety. (A) Funnel plot and Egger's test of vaccine efficacy. (B) Funnel plot and Egger's test of vaccine safety.

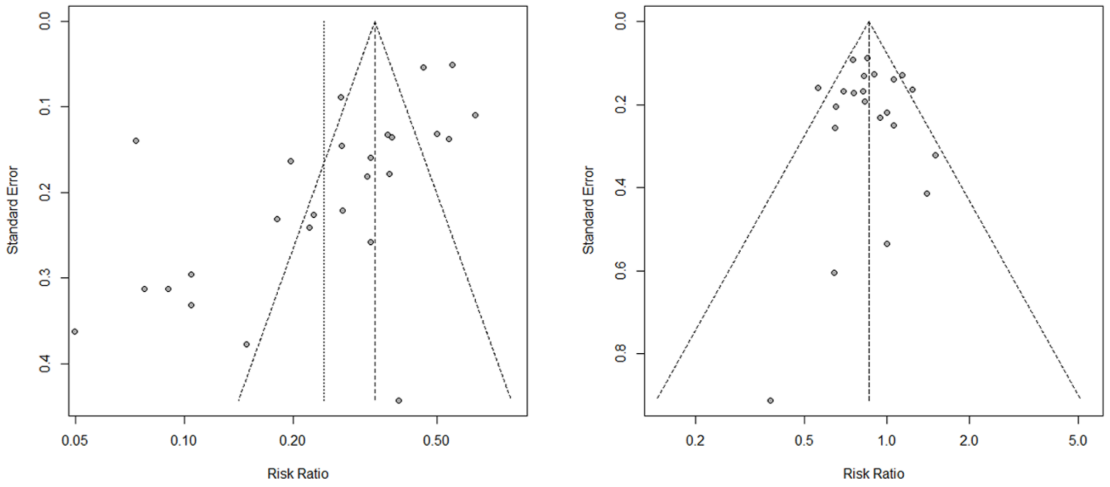

Egger' test: p = 0.0002
(A)

Egger' test: p = 0.7319
(B)

Table S8 Comprehensive comparisons for efficacy of individual vaccines in adults

**Placebo**																						
1.55 (1.26, 1.93)	**SpikoGen**																					
1.80 (1.65, 1.99)		**BIV1-CovIran**																				
1.86 (1.42, 2.44)			**CVnCoV**																			
2.18 (1.95, 2.41)	1.39 (1.09, 1.77)	1.20 (1.04, 1.38)		**Ad26-COV2-S**																		
2.39 (1.88, 3.00)	1.52 (1.11, 2.10)	1.31 (1.02, 1.70)			**CoronaVac**																	
2.69 (1.90, 3.82)	1.72 (1.14, 2.59)	1.48 (1.03, 2.12)				**Soberana**																
2.75 (2.12, 3.56)	1.75 (1.25, 2.46)	1.51 (1.14, 1.99)	1.46 (1.01, 2.14)				**Ad5-nCoV**															
3.06 (2.25, 4.22)	1.95 (1.35, 2.89)	1.68 (1.22, 2.34)	1.63 (1.08, 2.48)	1.39 (1.01, 1.95)				**SCB-2019**														
3.06 (1.88, 5.21)	1.97 (1.15, 3.49)	1.68 (1.02, 2.92)							**ZyCoV-D**													
3.10 (2.56, 3.78)	1.97 (1.49, 2.66)	1.70 (1.36, 2.12)	1.67 (1.19, 2.32)	1.42 (1.14, 1.77)						**AZD1222**												
3.13 (2.20, 4.53)	1.99 (1.32, 3.06)	1.72 (1.20, 2.51)	1.68 (1.07, 2.64)	1.43 (1.01, 2.10)							**CoVLP+ AS03**											
3.67 (2.41, 5.75)	2.34 (1.46, 3.86)	2.01 (1.31, 3.19)	1.97 (1.19, 3.35)	1.68 (1.09, 2.66)								**WIBP-CorV**										
3.67 (3.10, 4.39)	2.34 (1.79, 3.10)	2.01 (1.65, 2.46)	1.97 (1.42, 2.72)	1.68 (1.38, 2.05)	1.54 (1.15, 2.05)								**ZF2001**									
4.44 (2.89, 7.10)	2.83 (1.75, 4.71)	2.44 (1.57, 3.94)	2.39 (1.43, 4.10)	2.01 (1.31, 3.25)	1.86 (1.14, 3.13)									**BBV152**								
4.57 (2.89, 7.54)	2.92 (1.77, 5.00)	2.51 (1.57, 4.18)	2.44 (1.43, 4.31)	2.08 (1.31, 3.46)	1.92 (1.14, 3.32)										**BBIBP-CorV**							
5.05 (3.67, 7.03)	3.22 (2.20, 4.76)	2.77 (1.99, 3.90)	2.72 (1.79, 4.14)	2.32 (1.67, 3.25)	2.12 (1.42, 3.16)	1.88 (1.17, 3.00)	1.84 (1.22, 2.77)	1.65 (1.05, 2.56)		1.62 (1.12, 2.36)						**EpiVac Corona**						
5.53 (3.53, 8.76)	3.53 (2.14, 5.87)	3.03 (1.92, 4.90)	2.97 (1.75, 5.05)	2.53 (1.58, 4.06)	2.32 (1.39, 3.90)	2.03 (1.15, 3.67)	2.01 (1.20, 3.42)	1.80 (1.04, 3.13)		1.77 (1.08, 2.94)							**QazCovid-in**					
9.68 (6.36, 15.18)	6.17 (3.86, 10.18)	5.31 (3.46, 8.41)	5.21 (3.16, 8.76)	4.39 (2.89, 7.03)	4.06 (2.51, 6.75)	3.56 (2.08, 6.36)	3.53 (2.14, 5.93)	3.16 (1.86, 5.42)	3.13 (1.58, 6.11)	3.10 (1.95, 5.10)	3.06 (1.77, 5.42)	2.61 (1.42, 4.85)	2.61 (1.67, 4.26)	2.16 (1.16, 4.06)	2.12 (1.11, 4.01)	1.90 (1.12, 3.29)		**NVX-CoV2373**				
11.25 (6.23, 21.76)	7.17 (3.82, 14.44)	6.17 (3.39, 12.06)	6.05 (3.13, 12.30)	5.10 (2.83, 10.07)	4.71 (2.48, 9.49)	4.14 (2.10, 8.76)	4.10 (2.14, 8.33)	3.67 (1.88, 7.61)	3.63 (1.65, 8.33)	3.60 (1.93, 7.17)	3.56 (1.79, 7.54)	3.03 (1.45, 6.62)	3.03 (1.65, 6.05)	2.51 (1.20, 5.53)	2.46 (1.14, 5.47)	2.20 (1.13, 4.57)			**Gam-COVID-Vac**			
13.20 (7.46, 26.05)	8.50 (4.62, 17.29)	7.24 (4.06, 14.44)	7.10 (3.74, 14.73)	6.05 (3.39, 12.06)	5.53 (2.97, 11.36)	4.90 (2.51, 10.49)	4.81 (2.56, 9.97)	4.31 (2.23, 9.03)	4.31 (1.95, 9.87)	4.26 (2.32, 8.58)	4.22 (2.14, 9.03)	3.60 (1.73, 7.92)	3.60 (1.97, 7.24)	2.97 (1.42, 6.62)	2.89 (1.35, 6.49)	2.61 (1.35, 5.47)	2.39 (1.14, 5.37)			**Abdala**		
13.60 (10.49, 18.17)	8.76 (6.23, 12.43)	7.54 (5.64, 10.07)	7.32 (5.00, 10.80)	6.23 (4.71, 8.41)	5.70 (4.01, 8.25)	5.05 (3.25, 7.92)	4.95 (3.42, 7.32)	4.44 (2.94, 6.75)	4.44 (2.44, 7.85)	4.39 (3.16, 6.17)	4.35 (2.77, 6.82)	3.71 (2.20, 6.17)	3.71 (2.69, 5.16)	3.06 (1.8, 5.1)	2.97 (1.70, 5.10)	2.69 (1.77, 4.14)	2.46 (1.45, 4.18)				**mRNA-1273**	
20.91 (10.91, 46.99)	13.33 (6.69, 30.57)	11.47 (5.93, 25.79)	11.25 (5.53, 26.05)	9.49 (4.95, 21.54)	8.76 (4.35, 20.09)	7.69 (3.67, 18.54)	7.61 (3.74, 17.64)	6.82 (3.29, 16.12)	6.75 (2.92, 17.29)	6.69 (3.39, 15.33)	6.62 (3.13, 15.96)	5.64 (2.56, 14.01)	5.64 (2.89, 12.81)	4.71 (2.10, 11.59)	4.57 (2.01, 11.47)	4.10 (1.97, 9.68)	3.78 (1.68, 9.39)					**BNT162b2**

Data are RRs (95% CI) in the column-defining intervention compared with the row-defining intervention. RR higher than 1 favors the row-defining intervention. To obtain RRs for comparisons in the opposite direction, reciprocals should be taken. Results without statistical significance were deleted from the table.

Table S9. SUCRA value of various vaccines in the network meta-analysis of vaccine efficacy in adults.

Vaccine	SUCRA value
Placebo	1
SpikoGen	0.94
BIV1-CovIran	0.89
CVnCoV	0.87
Ad26-COV2-S	0.8
CoronaVac	0.74
Soberana	0.66
Ad5-nCoV	0.65
SCB-2019	0.58
ZyCoV-D	0.57
AZD1222	0.57
CoVLP+AS03	0.56
WIBP-CorV	0.46
ZF2001	0.45
BBV152	0.36
BBIBP-CorV	0.35
EpiVacCorona	0.3
QazCovid-in	0.28
NVX-CoV2373	0.15
Gam-COVID-Vac	0.12
Abdala	0.09
mRNA-1273	0.08
BNT162b2	0.02

Figure S5. Network diagram of individual vaccine safety in adults. The thickness of the lines is proportional to the number of trials comparing every pair of treatments.

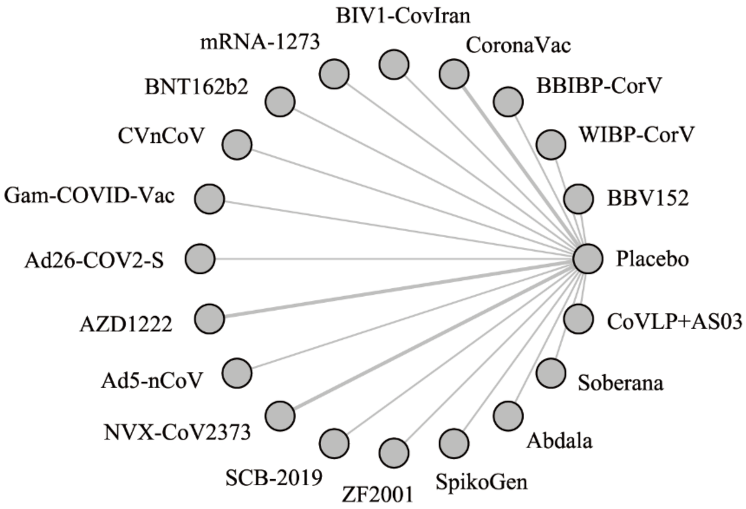

Table S10. Comprehensive comparisons for safety of individual vaccines in adults.

**CoVLP+ AS03**																			
	**CVnCoV**																		
		**Ad5-nCoV**																	
			**BNT162b2**																
				**mRNA-1273**															
					**Placebo**														
						**CoronaVac**													
							**SpikoGen**												
								**Abdala**											
									**NVX-CoV2373**										
	1.46 (1.01, 2.12)									**Ad26-COV2-S**									
											**SCB-2019**								
												**WIBP-CorV**							
1.93 (1.01, 3.82)	1.60 (1.08, 2.34)		1.46 (1.05, 2.03)		1.28 (1.04, 1.57)								**AZD1222**						
	1.65 (1.03, 2.64)													**BBIBP-CorV**					
1.99 (1.04, 3.90)	1.65 (1.14, 2.39)		1.51 (1.09, 2.05)	1.40 (1.01, 1.95)	1.32 (1.11, 1.58)										**ZF2001**				
2.29 (1.02, 5.21)	1.90 (1.03, 3.42)															**Gam-COVID-Vac**			
																	**Soberana**		
2.32 (1.09, 4.95)	1.92 (1.14, 3.22)		1.75 (1.08, 2.83)	1.63 (1.01, 2.69)	1.54 (1.03, 2.32)													**BBV152**	
2.66 (1.32, 5.47)	2.20 (1.40, 3.46)	2.51 (1.05, 6.17)	2.01 (1.35, 3.00)	1.90 (1.25, 2.86)	1.77 (1.30, 2.44)	1.75 (1.01, 3.06)			1.63 (1.12, 2.39)	1.51 (1.05, 2.14)									**BIV1-CovIran**

Data are RRs (95% CI) in the column-defining intervention compared with the row-defining intervention. RR higher than 1 favors the row-defining intervention. To obtain RRs for comparisons in the opposite direction, reciprocals should be taken. Results without statistical significance were deleted from the table.

Table S11. SUCRA value of various vaccines in the network meta-analysis of vaccine safety in adults.

Vaccine	SUCRA value
CoVLP+AS03	0.89
CVnCoV	0.84
Ad5-nCoV	0.82
BNT162b2	0.78
mRNA-1273	0.71
Placebo	0.67
CoronaVac	0.62
SpikoGen	0.58
Abdala	0.57
NVX-CoV2373	0.56
Ad26-COV2-S	0.44
SCB-2019	0.43
WIBP-CorV	0.41
AZD1222	0.34
BBIBP-CorV	0.32
ZF2001	0.3
Gam-COVID-Vac	0.22
Soberana	0.21
BBV152	0.2
BIV1-CovIran	0.1

Figure S6. Network diagram of type-specific efficacy in the elderly. The thickness of the lines is proportional to the number of trials comparing every pair of treatments.

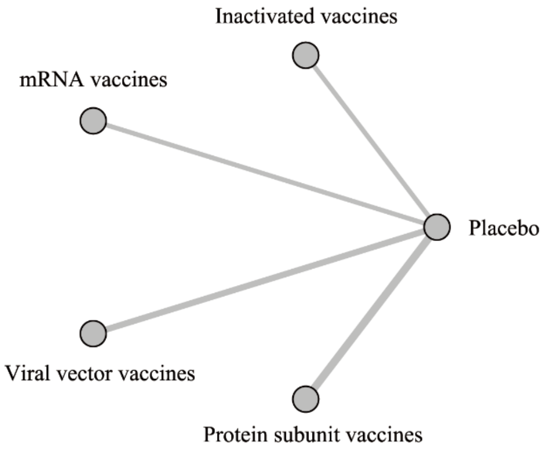

Table S12. Comprehensive comparisons for type-specific efficacy in the elderly.

**Placebo**				
	**Inactivated vaccine**			
4.35 (1.34, 13.87)		**Protein subunit vaccine**		
4.35 (1.43, 14.15)			**Viral vector vaccine**	
5.42 (1.49, 19.89)				**mRNA vaccine**

Data are RRs (95% CI) in the column-defining intervention compared with the row-defining intervention. RR higher than 1 favors the row-defining intervention. To obtain RRs for comparisons in the opposite direction, reciprocals should be taken. Results without statistical significance were deleted from the table.

Table S13. SUCRA value of various types of vaccines in the network meta-analysis of type-specific efficacy in the elderly.

Vaccine Type	SUCRA value
Placebo	0.98
Inactivated vaccine	0.6
Protein subunit vaccine	0.342
Viral vector vaccine	0.342
mRNA vaccine	0.24

Figure S7. Funnel plot and Egger's test of vaccine efficacy in the elderly.

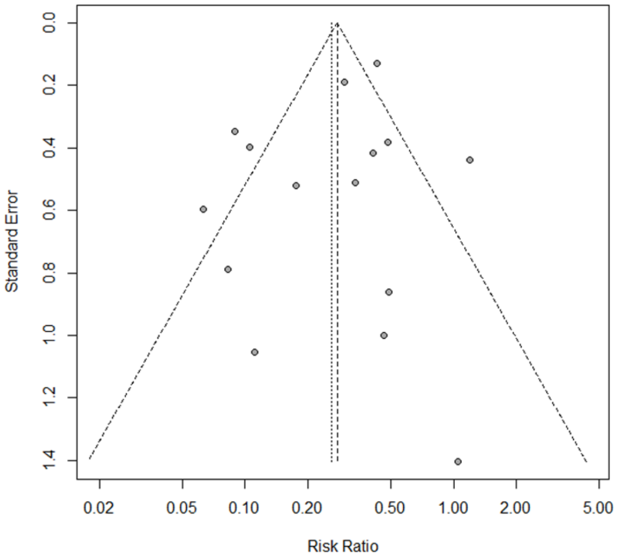

Egger' test： p = 0.2719

Figure S8. Network diagram of individual vaccine efficacy in the elderly. The thickness of the lines is proportional to the number of trials comparing every pair of treatments.

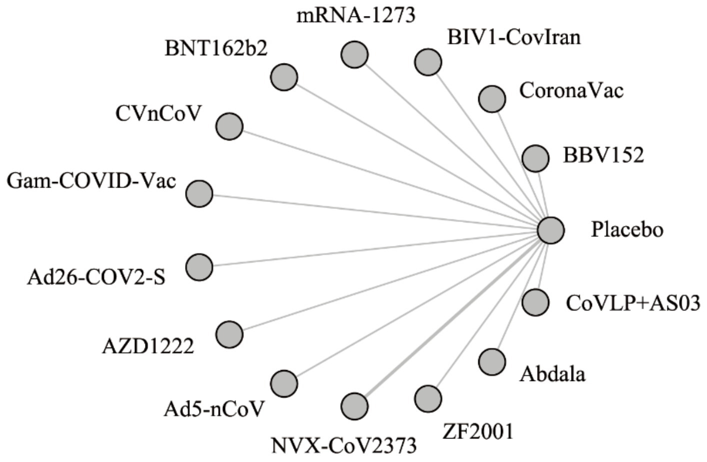

Table S14. SUCRA value of various vaccines in the network meta-analysis of vaccine efficacy in the elderly.

Vaccine	SUCRA value
CVnCoV	0.92
Placebo	0.9
CoVLP+AS03	0.76
Ad5-nCoV	0.68
BIV1-CovIran	0.66
CoronaVac	0.63
ZF2001	0.62
BBV152	0.54
Ad26-COV2-S	0.5
NVX-CoV2373	0.38
AZD1222	0.33
Abdala	0.19
mRNA-1273	0.15
Gam-COVID-Vac	0.15
BNT162b2	0.08

Table S15. Comprehensive comparisons for efficacy of individual vaccines in the elderly

**CVnCoV**														
	**Placebo**													
		**CoVLP+ AS03**												
	2.10<br>(1.01, 4.66)		**Ad5-nCoV**											
2.77<br>(1.13, 7.17)	2.32<br>(1.79, 2.97)			**BIV1-CovIran**										
					**CoronaVac**									
	2.48<br>(1.14, 6.05)					**ZF2001**								
3.74<br>(1.02, 15.80)	3.10<br>(1.19, 9.68)						**BBV152**							
4.01<br>(1.57, 10.80)	3.35<br>(2.34, 4.95)							**Ad26-COV2-S**						
6.23<br>(1.38, 36.60)	5.10<br>(1.52, 24.05)								**NVX-CoV2373**					
7.24<br>(1.92, 30.88)	5.99<br>(2.25, 18.92)									**AZD1222**				
12.06<br>(3.82, 42.10)	9.87<br>(4.81, 24.29)		4.71<br>(1.58, 14.88)	4.26<br>(1.97, 10.80)		3.97<br>(1.26, 12.94)		2.94<br>(1.30, 7.69)			**Abdala**			
14.01<br>(4.71, 44.70)	11.59<br>(6.17, 24.78)		5.53<br>(1.97, 15.80)	5.00<br>(2.51, 11.13)		4.66<br>(1.55, 13.74)	3.74<br>(1.02, 12.55)	3.46<br>(1.63, 8.00)				**mRNA-1273**		
16.78<br>(3.03, 149.90)	13.74<br>(3.25, 104.58)		6.55<br>(1.23, 56.26)	5.93<br>(1.36, 46.06)		5.47<br>(1, 47.94)							**Gam-COVID-Vac**	
21.54<br>(5.37, 119.10)	17.64<br>(6.23, 77.48)		8.50<br>(2.25, 42.95)	7.61<br>(2.59, 34.12)		7.10<br>(1.79, 37.71)	5.75<br>(1.21, 33.12)	5.26<br>(1.72, 24.05)						**BNT162b2**

Data are RRs (95% CI) in the column-defining intervention compared with the row-defining intervention. RR higher than 1 favors the row-defining intervention. To obtain RRs for comparisons in the opposite direction, reciprocals should be taken. Results without statistical significance were deleted from the table.

Table S16. Results of network meta-analysis after excluding trials with a follow-up time of less than 2 months (A) Type-specific efficacy in adults (B) Type-specific safety in adults (C) Type-specific efficacy in the elderly

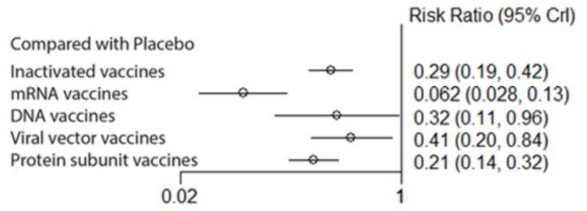

Vaccine Type	SUCRA value
Placebo	0.99
Inactivated vaccine	0.49
mRNA vaccine	0.00
DNA vaccine	0.54
Viral vector vaccine	0.69
Protein subunit vaccine	0.28

(A)

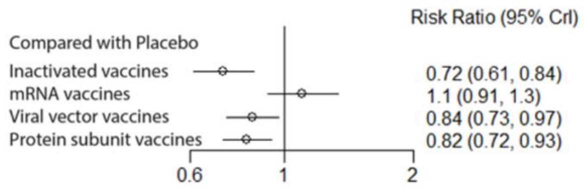

Vaccine Type	SUCRA value
Placebo	0.79
Inactivated vaccine	0.05
mRNA vaccine	0.96
Viral vector vaccine	0.39
Protein subunit vaccine	0.32

(B)

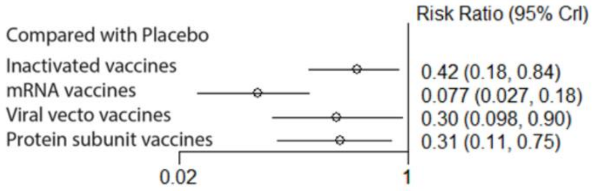

Vaccine Type	SUCRA value
Placebo	0.99
Inactivated vaccine	0.63
mRNA vaccine	0.01
Viral vector vaccine	0.42
Protein subunit vaccine	0.45

(C)
